# Postharvest practices, challenges and opportunities for grain producers in Arequipa, Peru

**DOI:** 10.1371/journal.pone.0240857

**Published:** 2020-11-04

**Authors:** Jorge R. Díaz-Valderrama, Anastasia W. Njoroge, Dennis Macedo-Valdivia, Nancy Orihuela-Ordóñez, Bradley W. Smith, Victor Casa-Coila, Nelly Ramírez-Calderón, Jackeline Zanabria-Gálvez, Charles Woloshuk, Dieudonne Baributsa

**Affiliations:** 1 Department of Entomology, Purdue University, West Lafayette, Indiana, United States of America; 2 Facultad de Agronomía, Universidad Nacional de San Agustín de Arequipa, Arequipa, Perú; 3 Facultad de Ingeniería Electrónica, Universidad Nacional de San Agustín de Arequipa, Arequipa, Perú; 4 Facultad de Psicología, Universidad Nacional de San Agustín de Arequipa, Arequipa, Perú; 5 Facultad de Ingeniería de Procesos, Universidad Nacional de San Agustín de Arequipa, Arequipa, Perú; 6 Department of Botany and Plant Pathology, Purdue University, West Lafayette, Indiana, United States of America; Hellenic Agricultural Organization - Demeter, GREECE

## Abstract

Little is known about the major issues leading to postharvest losses in Peru, which are estimated to be 15–27%. We surveyed 503 farmers from the lowlands and Andean regions of Arequipa to learn more about the major grains produced and issues encountered during drying and storage. Rice, common bean, and quinoa were the most grown crops in the lowlands while starchy maize was the most cultivated crop in the highlands. Most farmers (90%) dried their crops in-field directly on the ground, which exposes them to rodents, birds, and insect pests. The majority of farmers (92%) used subjective methods to assess grain moisture content. About 77% of farmers identified insects as a major challenge during storage but only 44% said they used preventive measures such as the application of insecticides. Among farmers who stored grain, the main reason was for household consumption (61%); while among those who did not store, the main reason was the need for immediate cash at harvest (75%). Farmers who experienced insect problems, who stored seed or grain for sale, who stored longer, or farmers from the lowlands were more likely to apply insecticides on their stored products. These findings provide an opportunity for researchers, development organizations, and government agencies to improve postharvest handling and storage in Arequipa by disseminating drying technologies, moisture assessment tools and hermetic storage solutions among farmers.

## Introduction

Peru agriculture is dominated by small-scale subsistence farmers who represent 74.7% of total farmers in the country [1, 2]. Their livelihood depends on crops produced on less than two hectares. Grown crops including common beans (*Phaseolus vulgaris* L.), quinoa (*Chenopodium quinoa* Willd.) and maize (*Zea mays* L.) are typically pre-dried in-field which exposes them to birds and rodents [3–5]. Once grain is dried and processed, farmers must decide whether to store or sell. However, only 6.4% of Peruvian farmers have adequate space for storage [2]. Postharvest losses are estimated between 15 to 27% due to limited training on proper grain handling and storage, and access to appropriate technologies [4, 6, 7]. Improving postharvest management practices would help mitigate losses along the crop value chains [8], and hence contribute to reducing poverty and food insecurity in the country.

In Peru, agricultural production areas are organized based on the national irrigation scheme, managed by the *Junta Nacional de Usuarios de los Distritos de Riego del Perú*, JNUDRP (Peruvian National Board of Water Users Associations of Irrigation Districts). Nationwide, there are 114 Water User Associations (WUAs) divided into 1,582 Irrigation Commissions (ICs) [9]. These organizations primarily manage water distribution and maintain the irrigation infrastructure. WUAs have developed strategic alliances with universities, Non-Governmental Organizations (NGOs), and other institutions to promote the development of local farming activities [10].

The department of Arequipa borders the Pacific Ocean and has several of these WUAs. Half of its territory is dominated by a broad arid coastal strip and the other half by the Andes highlands [11]. Arequipa’s agriculture is concentrated in the valleys and land along the rivers that make up the watershed basins originating in the Andes. “Camaná-Majes-Colca” is the largest basin in Arequipa and it irrigates the main grain producing areas in Arequipa: the Colca and Majes valleys, and the coastal province of Camaná [12, 13]. In the last three decades, irrigation projects (e.g. “Irrigación Majes”) have increased farm production in the arid areas by deviating waters from the “Camaná-Majes-Colca” basin [14–16].

Agricultural production in Arequipa includes quinoa, common bean, starchy maize, and rice (*Oryza sativa* L.). In the lowlands of Arequipa, most of these crops are produced by the WUAs. Arequipa is the highest producer of coastal quinoa in Peru [17] and is also the third highest common bean producing department [4]. In addition, rice and starchy maize are very important economic crops for the department of Arequipa. Though it contributes only 9% and 4.4% of the national rice and maize production, respectively, Arequipa yields are about twice the national average for both crops [18, 19]. Moreover, the Andean district of Cabanaconde is home of the multi-color “Cabanita” maize, culturally important for local consumers and in high demand by local, regional and national markets [20].

Most development efforts have gone into increasing crop production in the WUAs but little in improving postharvest management. A survey conducted in Peru showed that the national government and the private sector had invested little in research to improve rice production system but were interested in reducing postharvest losses [21]. This study estimated that rice suffers postharvest handling and storage losses between 6–10%, and field losses due to birds up to 20%. Another study in the departments of Lima and Huánuco estimated that postharvest losses of common beans ranged between 18% to 27%, and identified poor agronomic practices as major causes for these losses [4]. Little or no information is available on postharvest management of grain in the Arequipa department. Therefore, we conducted this study to assess the postharvest handling and storage of grains among farmers in the department. This information is needed to inform future interventions for improving crop production and value chains for smallholder farmers.

## Materials and methods

### Survey design

**Fig 1:**
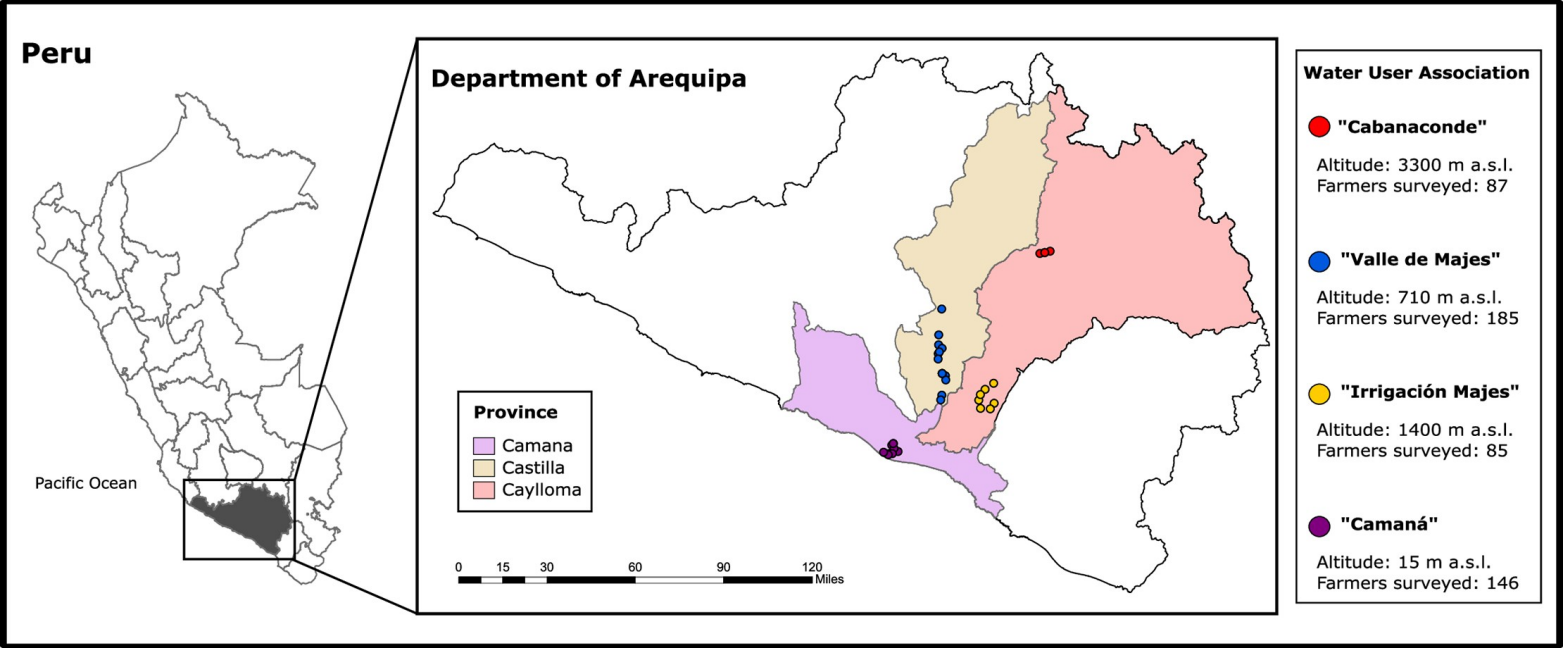
Map showing the Water User Associations (WUAs) in Arequipa, Peru where the study was conducted. Each dot represents one Irrigation Commission in each WUA.

This survey followed the best practices of international agricultural research and was approved under the Purdue University’s Institution Review Board # 1802020251 (ethics committee). The study was conducted in four WUAs that are among the main grain producing areas of Arequipa [12]: “Camaná” (CAM), “Irrigación Majes” (IM), “Valle de Majes” (VM) and “Cabanaconde” (CAB). Three of these WUAs (CAM, IM and VM) are located in the lowlands near the Pacific Ocean and below 1400 m above sea level (a.s.l.), while CAB is in the highlands at 3300 m a.s.l. in the Andean region (Fig 1). Fig 1 was generated in ArcGIS 10.6 (ESRI Inc., Redlands, CA, USA) by mapping the coordinates collected during the survey using a GPS locator feature embedded in the KoboCollect Application. The shapefiles of the administrative boundaries were obtained from the Environmental Territorial Information Platform of the Ministry of Environment of Peru (https://geoservidor.minam.gob.pe/). A total of 503 farmers were surveyed using a two-stage sampling design as described by Lumley (2010) [22]. First, we selected irrigation user commissions (ICs) within each WUA (Table 1). The number of ICs for each WUA was determined based on the proportion of total cultivated area for each main crop (Table 1). Based on these criteria we selected 12, 7, 15 and 4 ICs in CAM, IM, VM, and CAB, respectively, (Table 1). The ICs within each WUA were randomly selected with Microsoft Excel. The second step was to select a random sample of 12 farmers from each of the selected ICs (Table 1). The ICs memberships varied from as little as 12 farmers to as much as 500 farmers. For randomization purposes, we excluded all groups with less than 30 members. Farmers were invited to voluntarily participate in the survey and were asked to gather in their respective IC meeting location. If farmers failed to show up for the interview, they were replaced with other available farmers from the same IC. Replacements could not be found for three ICs (“Sonay” in CAM and “La Real” and “Querulpa” from VM), which resulted in only 11 farmers interviewed for each site. It is important to note that, based on the data and information available during sampling, rice was not considered as an important crop and hence was not used for assigning the number of respondents in this study. However, we collected data on rice in CAM and VM during the survey because of its importance in the production systems of farmers we interviewed.

**Table 1:** Total number of sampled Irrigation Commissions (ICs) and farmers interviewed in the four Water User Associations (WUAs) in Arequipa based on the cultivated area for a major crop.

WUA	Crop ^a^	Cultivated Area (ha) ^b^	Proportion (%)	Total ICs in a WUA	ICs ^c^ sampled	Farmers interviewed ^d^
Camaná	Common bean	1,040	30	17	12	146
Valle de Majes	Maize	1,325	39	17	15	185
Irrigación Majes	Quinoa	462	13	27	7	85
Cabanaconde ^e^	Maize	608	18	4	4	87
**TOTAL**		**3,435**	**100**	**65**	**38**	**503**

^a^ Rice was not considered in the original sampling strategy. However, we collected data on rice in Camaná and Valle de Majes during the survey because of its importance in the production systems of farmers we interviewed.

^b^ Source: Regional Agency of Agriculture, Arequipa [12]. Data from the 2016–2017 growing season.

^c^ Each ICs has a membership of 12 to 500 of farmers. For randomization purposes, we excluded all groups with less than 30 members.

^d^ Selected number of respondents in each WUA. We interviewed at least 12 respondents in each IC.

^e^ Cabanaconde is not a WUA but is a district in which four irrigation user commissions from the “Valle del Colca” IUB are located.

### Data collection

The questionnaire covered general demographic information, access to agricultural information, seed use, grain production and postharvest practices of four crops: rice, common bean (“Camanejo” cultivar), starchy maize (including “Cabanita” cultivar in the Andes), and quinoa. The questionnaire was uploaded into KoBoToolbox platform software (https://www.kobotoolbox.org) and loaded onto Android tablets for data collection [23]. All questions were closed-ended with only one answer to be selected. During the survey, trained and experienced enumerators speaking fluent Spanish administered the questionnaire. Before each interview, the enumerators explained to farmers the purpose of the study and anonymity of their responses. The enumerators then read a statement asking for farmer’s consent to participate in the interview. If the respondent agreed, the interview continued; otherwise the interview was stopped. Respondents were assigned a code, and no personal identifiers such as names, address, and phone numbers were collected during the study. Completed questionnaire forms were uploaded online daily for storage until analyzed.

### Data analysis

The data was downloaded in Microsoft Excel from KoBoToolbox and the raw data were manually curated for consistency and simplicity. The cleaned data were formatted and analyzed using the *R* package *survey* [24]. For the analysis, all questions were treated as categorical variables, except for the “size of household” and “duration of storage” which were numerical. Data points (individual surveys) were weighted by applying finite-population correction values (*fpc*), which for the first stage of the sampling design was the total number of ICs in each WUA and for the second stage was the total number of farmers in each IC. Results were presented in terms of WUA population percentage for categorical variables, and in terms of population mean for the numerical variables. Data on quantity of grain produced and stored were visualized in its unweighted form with boxplots.

Logistic regression models with the *glm* function in *R* v.3.5.3 were used to analyze the main factors that influence farmers’ decision to store grain and to use insecticides during storage. Independent variables used to assess farmers’ decision to store included “crop produced”, “size of household”, “education level”, “WUA”, “contact with extension agents”, and “quantity produced”. Farmers’ decision to use insecticide during storage was assessed with the independent variables “education level”, “reason to store”, “duration of storage”, “contact with extension agents”, “experienced insect problems” and “WUA’s altitudinal zone” (lowlands or Andes). The analyses were consistent with previous survey-based studies [25, 26]. To assess whether the logistic regression models were well fitted, we used the likelihood ratio (LR) test statistics [27]. To check for multicollinearity in the models, we assessed the correlations among variables with *R* package *psych* [28]. Since data analysis showed a strong correlation between “gender”, “reason to store” and “WUA’s altitudinal zone” in the model evaluating factors that influence the decision to use insecticides, we presented the results separately to avoid multicollinearity. In logistic regression analyses, the “quantity produced” data were transformed with the natural logarithm function. Outliers were not excluded for the logistic regression analysis. The threshold for significance was *p* = 0.05.

## Results

### Demographic characteristics of farmers

Most farmers were male (77.6%), married (72.8%) and 50 years or older (63.8%) (Table 2). Each household had an average size of four people. About 74% of the farmers had high school or tertiary (Institute or University) education. The primary economic activity was farming for the great majority of respondents (93.1%), while only 6.9% obtained most of their income from other activities such as private businesses, commodity trading and non-agriculture employment. Most farmers (78%) relied on their personal experiences for agricultural information including crop production and postharvest management.

**Table 2:** Demographic characteristics of farmers in the four Water User Associations (WUAs) in Arequipa.

Variables	Categories	Percentage (standard error)
**Gender (*n* = 503)**	Male	77.6 (2.12)
	Female	22.4 (2.12)
**Size of household (*n* = 503)**	3.9 (0.10)	
**Marital status (*n* = 503)**	Married	72.8 (2.27)
	Single	17.8 (1.86)
	Widow	5.6 (1.10)
	Divorced	3.8 (0.91)
**Age groups (*n* = 503)**	18–30	3.2 (0.73)
	31–40	16.2 (2.03)
	41–50	16.8 (2.07)
	>50	63.8 (2.21)
**Education level** ^a^ **(*n* = 503)**	None	2.2 (0.65)
	Primary	24.1 (2.07)
	Secondary	47. 6 (2.52)
	Tertiary	26.2 (2.23)
**Primary economic activity**^b^ **(*n* = 503)**	Farming	93.1 (1.27)
	Non-farming employment	3.6 (1.00)
	Commerce	2.6 (0.74)
**Primary source of agricultural information (*n* = 503)**	Personal experience	78.1 (2.16)
	Other farmers	8.2 (1.43)
	Agri-store specialist	7.1 (1.53)
	Extension agents	4.4 (1.03)
	Media	1.1 (0.52)
	NGOs	0.9 (0.40)

^a^ “Secondary” is equivalent to 1^st^-5^th^ year of High School in the Peruvian system; “Tertiary” is equivalent to having completed studies at an institution of higher learning such as an Institute or University.

^b^ “Non-farming employment” refers to jobs other than agriculture; “Commerce” means owning a business, trading or renting agricultural land. 0.7% of respondents noted that commerce was more profitable than farming.

### Crops grown, field handling, and production

The type and quantity of grain produced varied among the WUAs (Table 3). Rice was mainly produced in CAM (76%) and VM (71%); common beans mostly grown in CAM (86%); while starchy maize (Cabanita) was mostly grown in CAB (96%). Fewer farmers in both CAM (40%) and IM (11%) cultivated other varieties of starchy maize including purple maize. Quinoa was mostly produced in IM (70%) and to a lesser extent in CAB (44%). Improved varieties of quinoa were grown in the lowlands; while native quinoa ecotypes were cultivated in the highlands.

**Table 3:** Crops grown, average cultivated area and challenges during field drying in the four Water User Associations (WUAs) in Arequipa.

Variables	Categories	WUAs				Total
		CAM	IM	VM	CAB	
**Crops grown (%)**^a^		*n* = 146	*n* = 85	*n* = 185	*n* = 87	*n* = 503
	Rice	76 (4.4)^b^	0 (0.0)	71 (3.8)	0 (0.0)	47 (1.9)
	Common bean	86 (3.0)	7 (2.2)	8 (2.1)	0 (0.0)	35 (1.4)
	Maize	40 (5.4)	0 (0.0)^c^	11 (2.5)	96 (1.8)	25 (2.1)
	Quinoa	0 (0.0)	70 (5.2)	0 (0.0)	44 (5.3)	23 (1.5)
**Average cultivated area (ha)**		*n* = 142	*n* = 84	*n* = 182	*n* = 87	*n* = 495
	Rice	3.1 (0.57)	NA^d^	4.0 (0.50)	NA	3.4 (0.39)
	Common bean	2.4 (0.22)	1.5 (0.27)	2.0 (0.40)	NA	2.3 (0.19)
	Maize	1.4 (0.18)	NA	2.1 (0.53)	1.1 (0.16)	1.4 (0.13)
	Quinoa	NA	2.1 (0.20)	NA	0.6 (0.12)	1.9 (0.17)
**Challenges during field drying (%)**		*n* = 91	*n* = 42	*n* = 52	*n* = 47	*n* = 232
	Rodents	29 (6.1)	9 (3.9)	33 (7.1)	46 (7.2)	25 (3.5)
	Birds	11 (4.0)	44 (7.7)	30 (6.8)	26 (6.3)	24 (3.4)
	Fall to the ground	15 (4.0)	34 (6.3)	18 (5.6)	3 (3.2)	20 (2.7)
	Insect damage	11 (3.5)	12 (4.7)	16 (5.0)	15 (5.1)	12 (2.3)
	Molds	21 (4.6)	2 (2.3)	3 (2.5)	2 (1.8)	11 (2.4)
	Theft	14 (3.7)	0 (0.0)	1 (1.4)	9 (4.3)	8 (1.8)

^a^ Values do not add up to 100% as farmers may grow various crops.

^b^ Values are the estimated population percentages with standard errors in parentheses.

^c^ Farmers in IM cultivate maize for silage not for grain.

^d^ NA = Not applicable.

**Fig 2:**
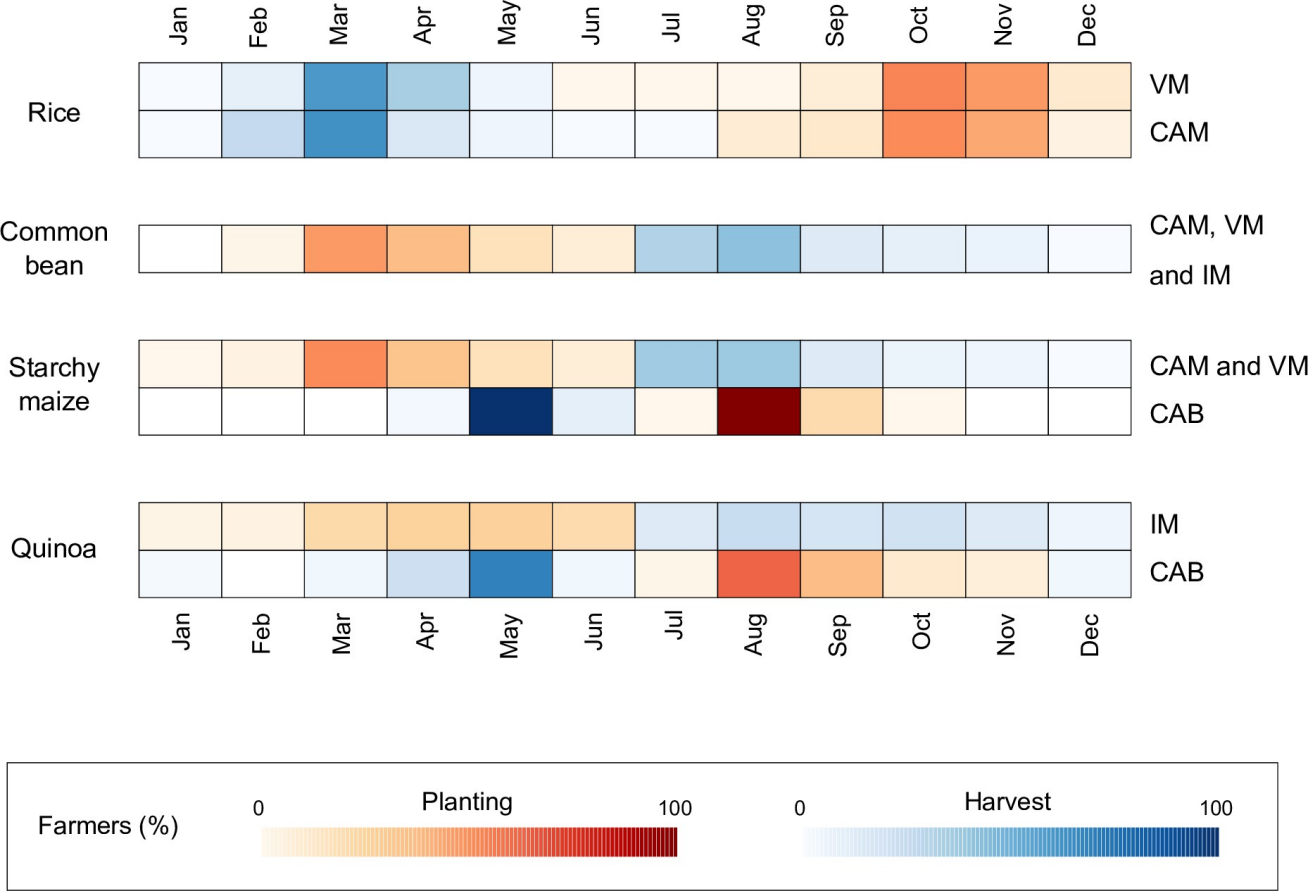
Main crop planting and harvesting seasons of the four Water User Associations (WUAs) in Arequipa. Heatmaps generated based on the cumulative responses of the respondents in each WUA.

Based on the survey responses, planting and harvesting seasons varied by crop and the altitudinal zones (Fig 2). Rice planting season in VM and CAM was between September and November, while the harvesting time was between February and April. In CAM and VM, most farmers planted a second crop (either common bean or starchy maize) after harvesting rice. These crops were harvested before the next rice planting season starting in September (Fig 2). Starchy maize in the lowlands (CAM or VM) took only four to five months to reach maturity. However, in the Andes (CAB), starchy maize (Cabanita) required nine months to reach maturity. Similarly, native ecotypes of quinoa in CAB were planted and harvested along with “Cabanita” maize, while improved quinoa varieties in IM had a shorter growing cycle (mostly planted in March–May and harvested after July).

Field drying crops before threshing or shelling was a very common practice among the interviewed farmers in Arequipa. Among farmers who field dried, 90.1% lay the harvested crop directly on the ground. Most farmers (83.6%) indicated that unfavorable weather was the major challenge during field drying. Furthermore, farmers noted that the most important sources of loss during field drying were rodents in CAM (29%), VM (33%) and CAB (46%), and birds in IM (44%). The second most important cause of loss were molds in CAM (21%) and grain shattering in IM (34%) (Table 3). After harvest, farmers continued to dry their grain at home. In VM, IM and CAM most farmers (84%, 91% and 96%, respectively) dried crops directly on the ground. However, in CAB, 52.6% of farmers used tarps or mats for drying mostly maize. Most farmers (92%) assessed dryness by subjective methods including biting, grain color, or the sound of grain when shaken. Only 8% of farmers noted that they use a moisture meter to assess grain dryness.

**Fig 3:**
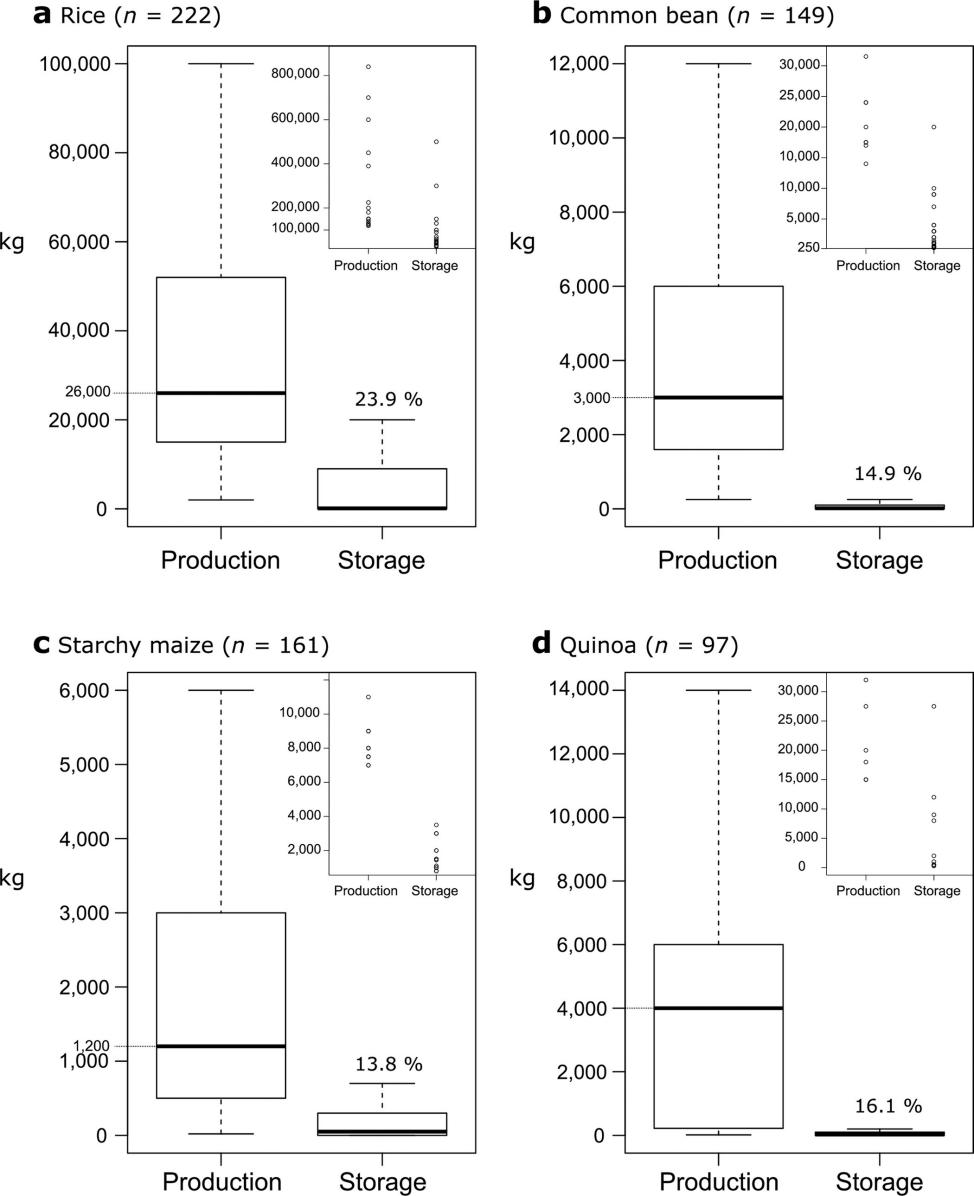
Grain produced and stored by the 503 respondents in the four Water User Associations (WUAs) in Arequipa. Smaller graph embedded in the main plot contains the outliers of each dataset; i.e. data points greater than the 75^th^ percentile value plus 1.5 times the interquartile range. Percentages correspond to the proportion of total quantity stored over total quantity produced including outliers.

Quantity of grain produced per farmer varied by crop: a median of 26,000 kg for rice (Fig 3A); 3,000 kg for common bean (Fig 3B); 1,200 kg for maize (Fig 3C); and 4,000 kg for quinoa (Fig 3D). The production of maize and quinoa varied with WUA location. The median maize production in CAB was 1,000 kg, while in CAM and VM was 1,750 and 4,600 kg, respectively. The median quinoa production obtained in CAB was 200 kg, while in IM was 5,250 kg (Fig 3).

### Storage capacity and management practices

Farmers storage capacity varied by crop and WUA (Fig 3). The proportions of total quantity stored over total quantity produced were 23.9% for rice, 16.1% for quinoa, 14.9% for common beans, and 13.8% for starchy maize (Fig 3). Among those producing rice, 57% stored with a 100 kg storage median (including farmers who did not store) (Fig 3A); while among farmers producing beans, 54.1% did store with a 15 kg total storage median (Fig 3B). Maize and quinoa storage varied depending on the WUA. Among farmers who produced maize, 98.8% stored in CAB (storage median of 225 kg), while 28.6% in VM and only 10.5% in CAM (storage median of 75 and 300 kg, respectively). Among farmers producing quinoa, those who stored were 97.4% in CAB (storage median of 55 kg) and 35.6% in IM (storage median of 100 kg) (Fig 3).

Farmers stored grain in different types of containers: most stored in polypropylene bags (89.8%) and the rest stored their grain in plastic drums or buckets (Table 4). Some farmers in VM and CAM used polypropylene fertilizer bags to store grain (Table 4). We also found farmers storing large quantities of paddy rice directly on the ground under a roof. In CAB, 19% of farmers described various types of containers, including metal pans, clay pots, and *trujes* (Table 4). When introduced to chemical-free hermetic storage technology (Purdue Improved Crops Storage—PICS bags), 91.5% of farmers said that they would be willing to purchase them. Farmers stored grain primarily for household consumption (61%) and for sale later in the year (31.7%) (Table 4). Securing grain for household consumption was more important in CAB than in CAM (72% vs 56% of farmers). Some farmers also stored seed for planting during the next season (Table 4). The majority of farmers (75.1%) said that the most important reason for not storing grains was the need for immediate cash at harvest (Table 4).

**Table 4:** Containers used, motivation to store, challenges, and protection during grain storage in the four Water User Associations (WUAs) in Arequipa.

Variables	Parameters	Water User Associations				Total
		CAM	IM	VM	CAB	
**Containers used to store grain**^a^ **(%)**		*n* = 122	*n* = 48	*n* = 152	*n* = 81	*n* = 403
	Polypropylene sacks	98 (1.2)	92 (3.2)	83 (3.5)	74 (4.9)	90 (1.4)
	Plastic drums/buckets/bottles	5 (1.9)	14 (4.3)	7 (2.3)	26 (4.9)	10 (1.4)
	Fertilizer plastic sacks	2 (1.4)	0 (0.0)	12 (3.1)	0 (0.0)	5 (1.1)
	Metal pans/Clay pots/*trujes*^b^	0 (0.5)	2 (2.1)	4 (1.6)	19 (4.2)	4 (0.8)
**Reason to store grain (%)**		*n* = 89	*n* = 28	*n* = 101	*n* = 85	*n* = 303
	Household consumption	56 (5.7)^c^	60 (9.3)	63 (5.3)	72 (4.8)	61 (3.3)
	For subsequent sale	37 (5.5)	32 (7.4)	28 (5)	23 (4.6)	32 (3.0)
	For seed	7 (2.8)	8 (4.7)	6 (2.1)	3 (1.6)	6 (1.6)
	Animal feed	0 (0)	0 (0)	3 (1.9)	1 (1)	1 (0.5)
	Barter	0 (0)	0 (0)	0 (0)	1 (1)	0 (0.1)
**Reason to not store grain (%)**		*n* = 53	*n* = 41	*n* = 80	*n* = 2	*n* = 176
	Immediate economic needs	60 (7.5)	80 (7.6)	84 (4.6)	100 (0)	75 (3.9)
	Not enough production	15 (5.4)	5 (2.9)	11 (3.8)	0 (0)	12 (2.9)
	Insect issues	25 (7.3)	5 (3.4)	5 (3.1)	0 (0)	10 (2.3)
	Good price at harvest	0 (0)	9 (4.2)	0 (0)	0 (0)	3 (1.7)
**Challenges during storage (%)**		*n* = 75	*n* = 23	*n* = 99	*n* = 81	*n* = 278
	Insect damage	75 (5.6)	66 (8.0)	92 (2.9)	64 (5.3)	77 (2.8)
	Rodents	18 (5.2)	26 (5.6)	6 (2.4)	35 (5.2)	18 (2.5)
	Molds	3 (2.1)	0 (0.0)	2 (1.6)	0 (0.0)	2 (1.0)
	Theft	2 (1.6)	4 (4.0)	0 (0.0)	1 (1.0)	2 (0.9)
	Grain quality reduction	2 (1.6)	4 (4.2)	0 (0.0)	0 (0.0)	1 (0.9)
**Protection methods during grain storage (%)**		*n* = 88	*n* = 28	*n* = 101	*n* = 85	*n* = 302
	Do nothing	42 (6.0)	82 (6.1)	49 (5.5)	77 (4.5)	56 (3.4)
	Insecticides	51 (6.0)	11 (5.2)	43 (5.4)	5 (2.4)	36 (3.2)
	Use of hermetic containers	4 (2.0)	7 (5.0)	5 (2.0)	2 (1.4)	4 (1.3)
	Chilling^d^	3 (2.4)	0 (0.0)	3 (1.7)	0 (0.0)	2 (1.1)
	Botanicals against insects	0 (0.0)	0 (0.0)	1 (0.6)	9 (3.2)	1 (0.5)
	Cats	0 (0.0)	0 (0.0)	0 (0.0)	4 (1.9)	1 (0.3)
	Rodenticides	0 (0.0)	0 (0.0)	0 (0.0)	3 (1.7)	0 (0.2)
**Reason to use insecticides (%)**		*n* = 37	*n* = 4	*n* = 44	*n* = 6	*n* = 91
	Effective	10 (5.5)	0 (0)	4 (3.9)	0 (0)	76 (5.1)
	Easy to use	18 (6.3)	0 (0)	10 (5.6)	0 (0)	14 (4.1)
	Available/Affordable	71 (7.6)	100 (0)	79 (7.3)	100 (0)	7 (3.4)
	Safe	1 (1.3)	0 (0)	7 (4)	0 (0)	3 (1.5)
**Reason to not use insecticides (%)**		*n* = 45	*n* = 23	*n* = 51	*n* = 75	*n* = 194
	Toxicity	58 (9)	60 (12.4)	75 (6.6)	86 (3.7)	68 (4.4)
	Do not have insect problems	20 (8.5)	35 (11.2)	7 (3.6)	2 (1.5)	16 (3.7)
	Not enough production	14 (5.4)	0 (0)	11 (4.3)	3 (1.8)	8 (2.0)
	Others^e^	9 (4.9)	5 (4.3)	8 (4.8)	8 (3.1)	8 (0.2)

^a^ Values add up to more than 100% since some farmers use different type of containers for storing grain.

^b^ A *truje* is a traditional conditioned space delimited with adobe bricks used for storage, only found in CAB.

^c^ Values are the estimated population percentages with standard errors in parentheses.

^d^ Farmers transport their grain to high altitude areas where temperatures are colder.

^e^ Insecticides are not effective, not available, or farmers do not know how to use them.

About three quarters of farmers (77%) indicated that insect damage was the greatest problem during grain storage followed by rodent attacks (18%) (Table 4). A little over half of farmers (56%) indicated that they did not take any action to mitigate these storage challenges (Table 4). This inaction was more predominant in IM (82%) and CAB (77%) where farmers did not protect their grain during storage (Table 4). Insecticide use was quite common in CAM (51%) and VM (43%) (Table 4). Among insecticide users, 76% of farmers considered them effective, while among non-users 68% of farmers noted toxicity as the main reason for not using them (Table 4). In CAB, farmers used traditional methods (e.g. botanicals) to control insects and cats and rodenticides against rodents. A small number of farmers used hermetic containers to protect their grain during storage. A few farmers (2%) used chilling methods by transporting their grain to high-altitude areas where temperatures are cooler (Table 4).

### Determinant of farmers’ decision to store and to use insecticides

Farmers’ decision to store grains varied by crops and the logistic regression models were found to be well fitted only for starchy maize and quinoa (significant LR test, *p*<0.001; (Table 5). Variable “quantity produced” was significantly associated with the decision to store maize (Table 5). As the quantity of maize produced increased (natural logarithm), the likelihood to store maize decreased (Odds ratio [OR] = 0.4; Table 5). Variable “WUA” was significantly associated with the decision to store in both maize and quinoa models. Education level, size of household and contact with extension agents did not associate with the decision to store. Some factors were found to influence farmers’ decision to use insecticide during storage and the logistic regression model was well fitted (LR test *p*<0.001; Table 6). The use of insecticides to protect stored grain was significantly influenced by the reason of storage (seed, home consumption, or for sale), the presence of insect pests, the zone (low or high lands), the duration of storage, and gender (OR significantly higher or lower than one for each of these variables; Table 6).

**Table 5:** Factors that influence farmers’ decision to store grains in the four Water User Associations (WUAs) in Arequipa.

Crop	Independent variable		OR^a^	95% CI^b^	*p*	LR test^c^
Rice (*n =* 222)	**Rice production**^**d**^		**1.4**	**[1.1, 2.0]**	**0.019**	
	Size of household		0.9	[0.8, 1.1]	0.542	
	*Education Level*	Basic/None/Primary	1.0	(referent)		*X*^2^ = 8.2
		High School	1.4	[0.7, 2.9]	0.308	*df* = 6
		Tertiary/University	1.3	[0.6, 2.9]	0.488	*p* = 0.225
	*Contact with extension gent*	No	1.0	(referent)		*LogLik* = −138
		Yes	0.9	[0.5, 1.7]	0.737	(*df* = 7)
	*Water User Association*	CAM	1.0	(referent)		
		VM	0.7	[0.4, 1.2]	0.172	
Common bean (*n =* 149)	Common bean production^d^		0.9	[0.6, 1.4]	0.775	*X*^2^ = 12.5
	Size of household		1.0	[0.8, 1.2]	0.886	
	*Education Level*	Basic/None/Primary	1.0	(referent)		
		High School	0.7	[0.3, 1.5]	0.381	*df* = 7
		Tertiary/University	1.9	[0.6, 6.2]	0.289	*p* = 0.086
	*Contact with extension agent*	No	1.0	(referent)		*LogLik* = −87.8
		Yes	1.5	[0.7, 3.4]	0.355	(*df* = 8)
		CAM	1.0	(referent)		
	*Water User Association*	IM	0.0	-	0.987	
		VM	1.3	[0.4, 4.2]	0.694	
Maize (*n =* 161)	**Maize production**^d^		**0.4**	**[0.1, 0.8]**	**0.017**	
	Size of household		0.9	[0.5, 1.3]	0.531	
	*Education Level*	Basic/None/Primary	1.0	(referent)		*X*^2^ = 115.1
		High School	2.6	[0.5, 16.6]	0.267	*df* = 7
		Tertiary/University	3.0	[0.4, 28.6]	0.309	*p*<0.001
	*Contact with extension agent*	No	1.0	(referent)		*LogLik* = −29.5
		Yes	0.3	[0.1, 1.5]	0.172	(*df* = 8)
	*Water User Association*	CAB	1.0	(referent)		
		**CAM**	**0.0**	**[0, 0.0]**	**0.000**	
		**VM**	**0.0**	**[0, 0.1]**	**0.001**	
Quinoa (*n =* 97)	Quinoa production ^c^		0.8	[0.3, 1.7]	0.502	
	Size of household		1.1	[0.7, 1.6]	0.628	
	*Education Level*	Basic/None/Primary	1.0	(referent)		
		High School	0.4	[0.1, 1.8]	0.259	*X*^2^ = 37.8
		Tertiary/University	0.8	[0.2, 3.4]	0.709	*df* = 6
	*Contact with extension agent*	No	1.0	(referent)		*p*<0.001
		Yes	1.0	[0.2, 40]	0.944	*LogLik* = −35.8
	*Water User Association*	CAB	1.0	(referent)		(*df* = 7)
		**IM**	**0.0**	**[0.0, 0.6]**	**0.049**	

^a^ OR = odds ratio. In bold are factors with ORs significantly different.

^b^ CI = confidence interval.

^c^ LR = Likelihood Ratio test; *X*^2^ = Chi-square value; *df* = degrees of freedom; *p* = probability value; *LogLik* = model’s log likelihood.

^d^ Production data was transformed with natural logarithm.

**Table 6:** Factors that influence farmers’ decision to use insecticides to protect their grain during storage in the four Water User Associations (WUAs) in Arequipa.

Independent variable	OR^a^	95% CI^b^	*p*	LR test^c^
*Education Level*				
Basic/None/Primary	1.0	(referent)		
High School	0.9	[0.4, 1.9]	0.746	
Tertiary/University	0.8	[0.3, 1.7]	0.503	
*Reason to store*				
Household consumption	1.0	(referent)		
**For subsequent sale**	**4.8**	**[2.4, 10.0]**	**0.000**	*X*^2^ = 85.5
**For seed**	**7.5**	**[2.3, 26.5]**	**0.001**	*df* = 10
Animal feed	0.0	-	0.986	*p* = 0.000
Barter	6.7×10^8^	-	0.989	
**Duration of storage**	**1.1**	**[1.0, 1.2]**	**0.049**	*LogLik* = −130 (*df* = 11)
*Contact with extension agent*				
No	1.0	(referent)		
Yes	1.7	[0.8, 3.3]	0.149	
*Experienced insect problems*				
No	1.0	(referent)		
**Yes**	**5.6**	**[2.3, 15.7]**	**0.000**	
*Altitudinal zone*^d^				
Highlands (Andes)	1.0	(referent)		
**Lowlands**	**12.7**	**[4.7, 41.5]**	**0.000**	
*Gender*^e^				*X*^2^ = 5.9; *df* = 1
Female	1.0	(referent)		*p* = 0.015
**Male**	**2.1**	**[1.1, 4.1]**	**0.02**	*LogLik* = −181.9 (*df* = 2)

^a^ OR = odds ratio. In bold are factors with ORs significantly different.

^b^ CI = confidence interval.

^c^ LR = Likelihood Ratio test; *X*^2^ = Chi-square value; *df* = degrees of freedom; *p* = probability value; *LogLik* = model’s log likelihood.

^d^ The water user associations CAM, IM and VM are in the lowlands, while CAB is in the Andes.

^e^ “Gender” has a significant correlation with the reason to store (either for household consumption or for sale, seed, animal feed and barter; *Pearson correlation value* = −0.14; *padj* = 0.01; *n* = 303); and altitudinal zone (*Pearson correlation value* = −0.12; *padj* = 0.01; *n* = 503). Therefore, its significance as a variable that influences the decision to store is presented in a separate model.

## Discussion

### Crop grown, field handling, and production

Rice is the most produced cereal in the country and Peru is the highest consumer in Latin America [29, 30]. Rice is mostly grown in the coastal region of Peru, with Arequipa (Camaná and the Majes Valley) being the most important producing department in the South [18, 30]. Maize is the second highest produced grain in the country, but most of it is yellow maize, which is used for animal feed [7]. Starchy maize, predominantly grown in the highlands, constitutes only 20% of the total maize produced in Peru [7, 29]. Both starchy maize “Cabanita” (grown in the highlands) and purple maize (cultivated in the lowlands) are of economic importance because they are used for human consumption. For instance, purple maize is used to produce a local beverage called *chicha morada*. Arequipa is also the third largest common bean producing department in the country. “Camanejo”, a bean cultivar developed in Camaná, is largely produced in Arequipa and found in markets nationwide [4, 31]. On the other hand, quinoa has historically been cultivated in the Andes. Over the last decade its cultivation has expanded to the coastal areas to meet the demand of international markets [17]. Currently, the lowlands of Arequipa supplies about 71% of the quinoa produced in the Peruvian coast [17].

Postharvest losses start to occur during field drying due to poor practices such as laying cut mature plants (maize or quinoa) on the ground. Farmers indicated that the drying process may take at least seven days when the weather is favorable. While field drying, crops were exposed to rodents, birds, insects, molds and thieves. Because of theft during field drying, some producers camped in their farms as a protective measure. The most reported problem during maize field drying in Cabanaconde were rodents and birds. A study on quinoa in Puno, in the Peruvian Andes with similar altitude to Cabanaconde, also found that birds were a major pest during field drying [5]. There is a need to introduce technologies that speed up the drying process in Arequipa. Drying technology such as EasyDry M500, developed for small farmers in Sub-Saharan Africa, would help reduce losses in Arequipa [32]. This portable dryer, with a 500kg capacity, can reduce maize moisture content from 20% to 13.5% in approximately three hours using cobs as the energy source [32, 33].

Arequipa farmers relied on subjective grain moisture indicators to assess dryness including feeling, color and sound. These methods of moisture assessment are commonly used in other parts of the world [34]. While over-drying grain can reduce potential profits due to loss of weight, under-drying can result in losses caused by fungi. A study in Honduras suggested that these subjective indicators contributed to 51% of samples being under-dried and 97% of the samples being contaminated with mycotoxins [35]. These mycotoxins can increase health risks to humans and livestock [36]. Grain moisture meters are expensive, and farmers are very unlikely to purchase them. However, over the last decade low-cost moisture assessment devices have been developed [37–40]. These devices either measure grain moisture content, estimate the water activity inside the grain or display the relative humidity and temperature at equilibrium which can be used to calculate moisture content. The use of these technologies in Arequipa would assist farmers in assessing when grain is dry enough for safe storage.

Data collected in Peru showed that more than 81 percent of harvested area along the coast was devoted to crops that were sold [41]. This is reflected in the quantity produced by farmers for each crop in Arequipa and demonstrates their economic importance. The market demand for crops such as rice and quinoa make them attractive cash crops among farmers in the coastal area of Arequipa. Crops such as quinoa had differential yields based on altitudinal zone (lowlands versus Andes/highlands). Cultivation in the lowlands requires a lot of inputs (fertilizers, pesticides, etc.), but yields can be up to three times higher than in the Andes, where agricultural production is mostly rainfed with minimal inputs [42]. A farmer in CAB who cultivated native ecotypes produced much less quinoa or starchy maize than a farmer in the lowlands who cultivated high-yielding varieties.

### Storage capacity and management practices

The need for immediate cash was the main reason farmers sold their grain right after harvest. The low proportion of total quantity stored over that produced suggest most farmers in Arequipa needed cash (e.g. to pay rental fees for the land used to produce the crop). Other farmers were not willing to take the risk of losing their harvest to insects during storage; and hence sold it. These results corroborate other findings that show most smallholder farmers in developing countries don’t store much grain because of need for cash at harvest to meet farming expenses and households’ needs [43]. Storing grain after harvest is mostly driven by the need to secure food for household consumption. Rice had the highest proportion of total quantity stored over total quantity produced because it is highly consumed and a very important food security crop in Peru [30]. These findings are consistent with results of studies conducted in developing countries in sub-Saharan Africa, the Middle East and South-East Asia [44–47].

Though insects were not a major issue during field drying, they become the most important challenge during storage, and were more prevalent on common beans and rice during storage in the lowlands. Warmer climate increased insect proliferation during storage. Farmers in the semi-arid eastern region of Kenya also noted that insects were their major issue during storage [47]. Rodent attacks were the second most important issue during storage after insects. Studies conducted in sub-Saharan Africa found that insects and rodents were major pests of stored maize and common beans [48, 49].

### Determinants of farmers’ decision to store and to use insecticides

Farmers in the lowlands (CAM and IM) were less likely to store maize or quinoa compared to farmers in the highlands (CAB). Similar results on storage have been reported when comparing quinoa production in “Irrigación Majes” in Arequipa and Camacani in Puno, Peru, at 3,800 m a.s.l. [42]. Our survey also revealed that some farmers from the lowlands transported their harvested grain to the Andean region for storage to reduce insect damages. Farmers in CAB complained that quinoa from the lowlands, stored in the highlands, was being marketed as Andean quinoa. We suggest interventions that provide crop traceability to protect the market of Andean quinoa. For maize, the significant association found between the quantity produced and the decision to store indicated that farmers producing more maize were less likely to store. This observation is in agreement with a report stating starchy maize (e.g. purple maize in the lowlands) is a cash crop that is mostly sold right after harvest [7].

Findings from our survey indicated that farmers who stored seed or grain to sell, and stored for longer periods, were more likely to use insecticide. Similar results have shown that farmers who stored grain for sale in Nigeria, those who stored seed in Mexico and Ethiopia, and those who stored longer in Benin were more likely to use insecticides [50–53]. Farmers in the lowlands, especially those storing rice and common beans, were more likely to use insecticides. The fact that less female farmers were likely to use insecticides than male farmers may be related to food safety concerns. Some farmers indicated that they did not use insecticides because of toxicity. A study conducted in Kenya found that women farmers were significantly more likely to use plant extracts (e.g. botanicals) to control storage pests [54].

Farmers were interested in chemical-free storage technologies. When introduced to hermetic PICS bags, most farmers expressed willingness to purchase this technology. In Kenya, the elimination of insecticide use (health benefits) is one of the most important reasons farmers are using hermetic storage methods [55]. There are several hermetic technologies that could be promoted to farmers in Arequipa including metal silos and hermetic bags [56–59]. Hermetic technologies have shown to be effective against insect pests, improve food security, income, and welfare of farmers [59–63]. When disseminating hermetic storage technologies in Arequipa, there is a need to include moisture assessment tools; high grain moisture content leads to fermentation under hermetic conditions [64].

## Conclusion

Arequipa is a very important grain producing department in Peru. This study identified several challenges during: i) field drying (drying on the ground, bad weather and pests), ii) grain moisture assessment (use of subjective methods), and iii) storage (pests and use of insecticides). Postharvest handling and storage losses are important in Arequipa but more acute in the lowlands. Insects were the major pest of stored grains with rice and common beans being the most susceptible during storage. Our findings provide valuable information to development and government agencies that are interested in helping farmers improve food security in both lowlands and highlands of Arequipa. Postharvest interventions through trainings and demonstrations should be tailored for each altitudinal zone and include dryers, low-cost moisture assessment devices, and hermetic containers.

## Supporting information

S1 Table Data collected from grain farmers in four Water User Associations from Arequipa. (XLSX)
